# Medical Education in Japan

**Published:** 1904-12

**Authors:** 


### MEDICAL EDUCATION IN JAPAN.*

It was about the year 1771 that a Dutch text-book of anatomy came into the hands of a Japanese physician. He and three of his friends, seeing from the illustrations that either their teaching had been wrong or the teachings of the Dutch text-book were false, arranged with an executioner to dissect the body of a criminal and so satisfy themselves as to who was right. They found that the Dutch authority was correct, and so they set to work to decipher the text. One of these men knew a few Dutch words, the others were entirely ignorant of the language. Taking as a starting-point a few words such as "head," "face," "hand," etc., found in connection with the illustration, these men worked daily at the translation until, at the end of four years' hard work they were able to read about half a page in an afternoon ; but they persisted, and after rewriting the whole book eleven times, they were able to publish a good translation. This was the first western knowledge introduced into the Japanese empire. Later came chemistry, natural history, history, military tactics, etc.

---

*An address delivered by Professor Kakichi Mitsakuri, Imperial University of Japan, at the St. Louis Medical Society, October 1, 1904.

In 1868 the Imperial University was established. It has a four-years' course, admitting one hundred students to each course.

There are three grades in the educational system of Japan, the university, the professional school, and all the schools below that grade. The boy begins going to school at six years. At twelve he goes to the middle school (a little lower than the American high school). In that school he spends five years and then passes into the higher school, where he spends three years. The higher school prepares directly for the university, where he spends four years. In the high school he studies one foreign language, either English, French, or German, English generally being taken. Those who plan to become physicians also take German, for German is the medical language of Japan. The medical profession is hereditary in Japan, so that many youths at twelve or thirteen years of age take up German, with the intention of being physicians. All physicians in Japan speak German, and many of them write it.

The arrangement of the course is based on that of the German schools and at one time all professors were German. Now all the professors are Japanese, except two honorary members of the faculty. The subjects studied in the medical college are similar to those studied in American colleges. There are two examinations. At the first the student is required to pass in anatomy, physiology, chemistry, pharmacology, general pathology, etc. At the final examination he is given surgery, obstetrics, laryngology, otology, ophthalmology, and gynecology (hygiene, forensic medicine, pediatrics, and syphilis being among the elective studies).

Attached to the university is a hospital having about four hundred beds. Hundreds of patients go to the hospital in a day, and it is a great problem how to limit the number, so that the students have an excellent opportunity for bedside instruction.

After the student receives his degree at the end of four years he is allowed to practice without any further examination, but many students remain for three or four years more study and then are sent to Germany to complete their medical education; so that by the time a man has completed his medical studies he is usually nearly thirty years old, but he is supposed to have received the highest training possible.

A number of easier schools have been established in Japan where students are allowed to go directly from high school. In these professional schools the Japanese language is used but they turn out a very good class of physicians. The graduates of these professional schools are also allowed to practice without a State examination.

Then comes the third class of students. They go through an irregular course of training. These men are allowed to obtain a license by going through a State examination. There are two classes of State examinations. The first covers the ground work, anatomy, physiology, etc. After they have passed that, they are permitted to take the second examination, covering internal medicine. The demand for physicians in Japan is not completely supplied, especially in the country districts, but in a few years the number of physicians will be adequate to all requirements.—*St. Louis Medical Review.*

